# A Novel Pharmacological Method to Study the Chinese Medicinal Formula Hua-Zheng-Hui-Sheng-Dan

**DOI:** 10.1155/2015/436807

**Published:** 2015-09-01

**Authors:** Rui Cao, Hong Zhang, Jie Guo, Xiao-hui Liu, Chang Liu, Cui-hong Zhu, Xiong-zhi Wu

**Affiliations:** ^1^Zhong-Shan-Men Inpatient Department, Tianjin Medical University Cancer Institute and Hospital, National Clinical Center for Cancer, Key Laboratory of Cancer Prevention and Therapy, Tianjin 300171, China; ^2^Department of Head and Neck Cancers, West China Hospital, Sichuan University, Chengdu 610041, China; ^3^Chengdu Diao Tianfu Pharmaceutical Group Co., LTD, Chengdu 610041, China

## Abstract

*Objectives*. Hua-Zheng-Hui-Sheng-Dan (HZHSD) was used as an experimental model to explore research methods of large formulae in traditional Chinese medicine (TCM) using current molecular biology approaches. *Materials and Methods*. The trypan blue exclusion assay was used to determine cell viability and cell numbers. Flow cytometry was used to assess cell cycle distribution and apoptosis. The concentration of cyclin D1 was analyzed by enzyme-linked immunosorbent assay. The median effect principle was used in drug combination studies. An orthogonal experimental design was used to estimate the effects of each herb at different concentrations. The HeLa xenograft mouse model was used to compare the antitumor activity of drugs in vivo. *Results*. Among the 35 herbs that comprise HZHSD, Radix Rehmanniae Preparata (RRP), *Caesalpinia sappan* (CS), *Evodia rutaecarpa* (ER), Folium Artemisiae Argyi (FAA), *Leonurus japonicus* Houtt (LJH), Tumeric (Tu), Radix Paeoniae Alba (RPA), and Trogopterus Dung (TD) effectively inhibited the proliferation of HeLa and SKOV3 cells. Only RRR had an effect on HeLa and SKOV3 cell viability. According to the median effect principle, *Angelica sinensis* (Oliv.) (AS), *Tabanus* (Ta), and Pollen Typhae (PT), which were proven to have a significant synergistic inhibitory effect on the proliferation of HeLa cells, were added to the original eight positive herbs. The combination of RPA and AS had a synergistic effect on inducing cell cycle S phase arrest and decreasing intracellular cyclin D1 in HeLa cells. By orthogonal experimental design, LJH and Tu were considered unnecessary herbs. The small formula (SHZHSD) consisted of RPA, AS, RRR, Ta., TD, PT, ER, CS, and FAA and was able to inhibit cell proliferation and induce cell apoptosis. The antitumor effects of HZHSD and SHZHSD were also compared in vivo. *Conclusions*. Through molecular biology approaches both in vitro and in vivo, research into single drugs, and analysis using the median effect principle and orthogonal experimental design, the small formula (SHZHSD) was determined from the original formula (HZHSD). SHZHSD exhibited superior antitumor activity compared with the original formula both in vitro and in vivo.

## 1. Introduction 

With the development of researches for the pathogenesis of diseases, combination therapies exert more advantageous effect than single-drug therapy [1]. In addition, the big change of the treatment principle showed that combination therapy occupies a more and more important position in the clinical application of disease treatment. In particular for tumor, combination therapies are commonly used for the reason of complex pathogenesis and various clinical manifestations of tumor. For example, not only are the therapeutic methods such as operation, radiotherapy, chemotherapy, biotherapy, and traditional Chinese medicine (TCM) therapy diversified but also the specific drugs of chemotherapy and TCM are multiple. Combination medicines with multieffect pathways and targets present better effect than single drugs [2]. There is a long history of the TCM applied for tumor therapy. Moreover, formulae, which are composed of the principal components and several adjuvant components, are the important constitution of TCM [3, 4]. However, a large number of formulae consisted of dozens of herbs or minerals. The mechanisms and the main active ingredients of these formulae still need to be discovered by using molecular approaches. Thus, compared with other small formulae, there is a greater difficulty in such a study.

For example, Realgar-Indigo naturalis formula is one classical small formula which is composed of Realgar, Indigo naturalis, and* Salvia miltiorrhiza*. It was found that it had a good effect on treating promyelocytic leukemia [3]. Another typical small formula is Huangqin-Tang, which is composed of* Scutellaria baicalensis*,* Paeonia lactiflora*,* Glycyrrhiza uralensis* and jujube, which has been found to relieve the intestinal injury caused by chemotherapy [5]. In addition, studies of the antitumor effect of single herbs or monomers in medicinal herbs are relatively new in recent years. For example,* ginseng*,* Curcuma longa,* and* Mylabris* have been found to have a potentially positive effect on treating cancer [6–8]. We can apply these previous research methods to the study of large formulae. By screening every herb in a formula, we can determine which herbs have antitumor activity. In doing so, the large formula can be reduced to a smaller formula.

Hua-Zheng-Hui-Sheng-Dan (HZHSD) is a classic formula in TCM that was recorded in* Wenbingtiaobian* by Wu Tang during the reign of Emperor Qianlong. It is one of the most famous Chinese herbal formulae used to treat malignant tumors. Both the tablet and oral liquid forms of HZHSD were approved by the State Food and Drug Administration (SFDA) as antitumor drugs [9].

**Figure 1:**
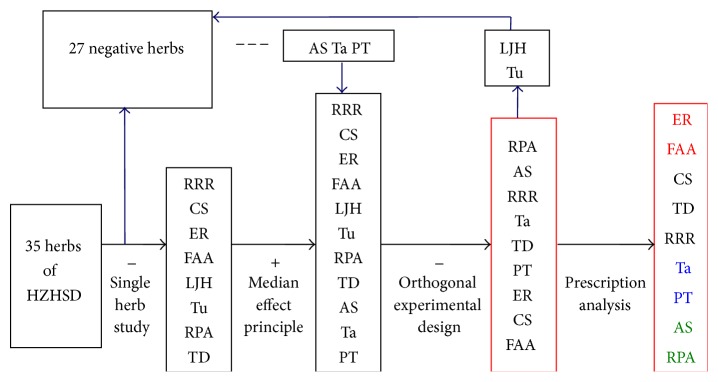
Technology roadmap of our research.

However, there are 35 different herbs in the large formula (Table 1) [10]. The clinical application of HZHSD was restricted to a certain extent due to the multiple ingredients. Therefore, in trying to investigate the possible research method of large formulae in TCM with current molecular biology approaches, we use the treatment human cervical cancer HeLa cells with HZHSD as the working model. Through in vitro and in vivo experiments, the median effect principle, and orthogonal experimental design, we were able to optimize and reduce the large formula (Figure 1).

To supplement the missing and synergistic herb after single herb screening, our study applied the median effect principle to research important combinations in the formula. The median effect principle is widely used in drug combination studies. It provides quantitative definitions for antagonism, additive effect, and synergism in drug combinations [11, 12]. Orthogonal experimental design, also called Taguchi method design, studies a large number of variables with a small number of experiments by orthogonal arrays [13]. According to orthogonal arrays, the number of experimental configurations was significantly reduced. Moreover, the conclusions obtained from small-scale experiments exerted better validity than the entire experimental region [13, 14]. Therefore, this statistical procedure could be used to analyze the decomposed recipes of large formulae. It estimates the effect of a single herb at different concentration levels by utilizing the tables of orthogonal arrays and analysis of variance (ANOVA) to [15, 16]. By analyzing the data, we can determine the optimal levels of each component and exclude unnecessary drugs.

## 2. Materials and Methods

This study was performed with the approval of the Animal Ethical and Welfare Committee of Tianjin Medical University Cancer Institute and Hospital (Approval number: 019).

### 2.1. Herbs and Preparation of Aqueous Extracts

All herbs in the formula were obtained from the Manufacturing and Distributing Branch of the Sichuan Chinese Herb Company on January 5, 2011. A voucher specimen was deposited in the Zhong-Shan-Men Inpatient Department of the Tianjin Medical University Cancer Institute and Hospital.

Whole herbs were ground into powder and extracted twice with hot distilled water (first with 1000 mL and next with 800 mL) at 100°C for 1 or 0.5 hours, respectively. The solutions were combined and centrifuged at 5000 rpm for 10 minutes. The resulting supernatant was filtered, concentrated, and dried to obtain a residue. The residue was dissolved in phosphate buffered saline at the desired concentration and stored at −20°C as the stock extract solution.

### 2.2. Reagents, Cell Lines, and Mice

The HeLa cervical cancer and SKOV3 ovarian cancer cell lines were obtained from Tianjin Medical University Cancer Institute and Hospital. Cells were allowed to grow for 24 hours in DMEM medium (Hyclone, USA), respectively, supplemented with 10% fetal bovine serum (Hyclone, USA) before being exposed to drugs. Female nu/nu nude mice were obtained from the Chinese Medical Academy of Science and used according to the National Institutes of Health guidelines for animal care. All surgeries were performed under sodium pentobarbital anesthesia, and every effort was made to minimize suffering. All in vivo studies were performed in the Centre Laboratory of Tianjin Medical University Cancer Institute, which is the authentic Animal Laboratory of the Committee on the Ethics of Tianjin Medical University Cancer Institute and Hospital. The enzyme-linked immunosorbent assay (ELISA) kit was purchased from Dongge (China). FITC, annexin V, and propidium iodide (PI) were purchased from Becton Dickinson Biosciences (USA).

### 2.3. Trypan Blue Exclusion Assay

HeLa and SKOV3 cells were seeded in 24-well plates at 0.5 × 10^4^ cells/well and 1 × 10^4^ cells/well, respectively. Cells were collected at 96 hours after treatment with drugs. The trypan blue exclusion assay was used to determine the viable and total cell numbers. The concentration of herbs was calculated using the following equation: *C* (*μ*g/mL) = 5 × *D* (mg/Kg/d), in which *D* is the clinical drug concentration. In this study, according to the proportion of each herb's dose in HZHSD, we selected 6 g as the basal clinical drug dosage of every herb.

We found three smaller formulae of HZHSD: Si Wu Tang (AS, RPA, LCH, and RRP), Di Dang Tang (RRR, Ta, SP, and Le), Shi Xiao San (TD and PT). The dose-effect curves of single or combined drug treatments were analyzed using the median effect principle studied by Chou and Talalay [11, 12]. For the fraction affected (Fa), 1 is equivalent to 100% inhibition. The combination index (CI) was calculated using the CI-isobologram method based on data derived from cells treated with the single drugs and drug combinations at the indicated weight ratios and plotted versus Fa. CI values were calculated using the following equation: CI = (*D*)1/(*Dx*)1 + (*D*)2/(*Dx*)2. (*Dx*)1 and (*Dx*)2 are the concentrations of one drug and the other alone at *x*% growth inhibition. (*D*)1 and (*D*)2 were the concentrations of the drugs in combination at *x*% growth inhibition. (*Dx*)1 and (*Dx*)2 were calculated using the median effect equation: *Dx* = *Dm*[Fa/(1 − Fa)]1/*m*. *Dm* is the median effect dose, and *m* is the slope of the median-effect plot [17].

The Taguchi method primarily relies on the systematic approach of conducting a minimum number of experiments using the mathematical instrument known as orthogonal arrays (OA) [10]. The method is primarily used to achieve the contribution of each variable and its level to obtain an optimum combination. The method also gives a full description of all the factors that affect the performance parameters. Based on the number of factors and their levels, L18 (5^3^) orthogonal array was applied [15, 18]. The five positive herbs were the five factors. All factors were assigned three levels, that is, low dose, high dose, and control group.

### 2.4. Flow Cytometry Analysis

HeLa cells were seeded at 3 × 10^4^ cells/well in 6-well plates. Cells were collected 96 hours after adding positive drugs. To detect apoptosis in the early stage and later stage, cells were stained with FITC and annexin V and PI, respectively. For cell cycle analysis, cells were fixed with 75% alcohol and stained with PI alone (50 *μ*g/mL). Apoptosis and DNA distribution were analyzed by flow cytometry (BD FACSCalibur, USA).

### 2.5. ELISA

HeLa cells were seeded at 3 × 10^4^ cells/well in 6-well plates. After 24 hours, the medium was replaced with positive drug-containing medium, and the cells were incubated for additional 96 hours. The cell monolayers were lysed with 100 *μ*L of RIPA buffer for 30 minutes, collected, and centrifuged at 12000 rpm for 5 minutes at 4°C. The supernatants were collected, and the concentration of cyclin D1 was detected according to the manufacturer's protocol.

### 2.6. Tumor Xenograft Assay

Female nu/nu nude mice, aged 4 to 6 weeks and weighing approximately 18–20 g, were injected subcutaneously with HeLa cells (0.1 mL, 1 × 10^6^). The mice were allocated into three groups according to the following treatment: (1) normal saline (control group), (2) 6 g and 18 g large formula clinical drug concentrations, and (3) 6 g and 18 g small formula clinical drug concentrations. The clinical drug concentrations refer to the dosage applied to human. The administration of the drug began the day after the inoculation, and each animal received the drug once a day for 22 days via cavitas abdominalis injection. Each tumor was measured every three days in two dimensions (length and width) using calipers, and the tumor volume (mm^3^) was calculated (*V* = length × width^2^/2). On the 25th day, animals were weighed and sacrificed. The implanted tumors were excised and weighed.

### 2.7. Statistics and Data Analysis

All experiments were repeated three times, with three replicate samples in each experiment. Single-factor analysis of variance was used for data analysis. Statistical calculations were performed using SPSS (version 16.0, Chicago, USA).

## 3. Results

### 3.1. Effects of a Single Herb of HZHSD on Cell Proliferation

**Figure 2:**
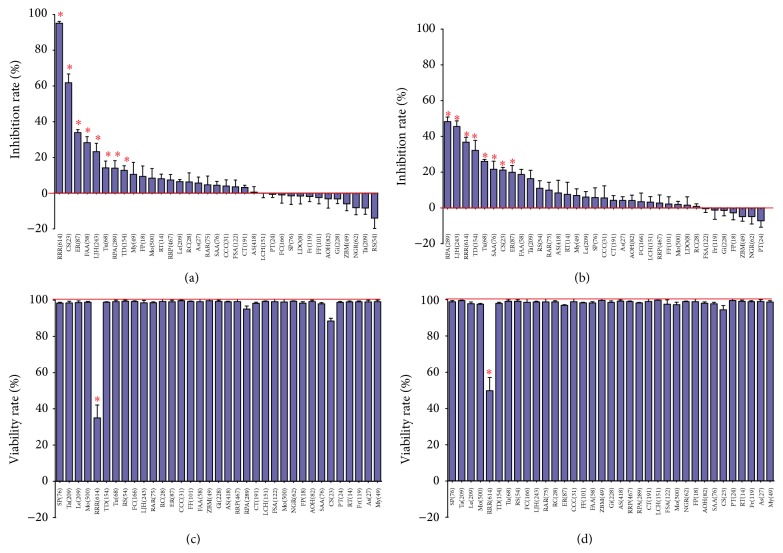
Effect of single herbs of HZHSD on cell proliferation. (a) Inhibitory rates of HeLa cells treated with single herbs of HZHSD. (b) Inhibitory rates of SKOV3 cells treated with single herbs of HZHSD. (c) Viability rates of HeLa cells treated with single herbs of HZHSD. (d) Viability rates of SKOV3 cells treated with single herbs of HZHSD. ^*∗*^ *P* < 0.05 versus the control group. Drug dose unit: *μ*g/mL.

The antiproliferation effects of every herb in HZHSD were investigated using the trypan blue counting method. Our data showed that RRR, CS, ER, FAA, LJH, Tu, RPA, and TD effectively inhibited the proliferation of HeLa cells and SKOV3 cells (*P* < 0.05). The maximum inhibition rates for RRR 614 *μ*g/mL and CS 23 *μ*g/mL on HeLa were 95.03% and 61.75%, respectively. Further, 289 *μ*g/mL RPA effectively inhibited the proliferation of SKOV3 cells. The inhibition rate was 48.25%. Additionally, 76 *μ*g/mL SAA only could inhibit the proliferation of SKOV3 cells. The inhibition rate was 21.63% (Figures 2(a) and 2(b)). Among the 35 types of herbs in HZHSD, only RRR had an effect on HeLa and SKOV3 cell viability (Figures 2(c) and 2(d)).

### 3.2. Median Effect Principle Used to Study the Effect of Si Wu Tang, Di Dang Tang, and Shi Xiao San on HeLa Cell

**Figure 3:**
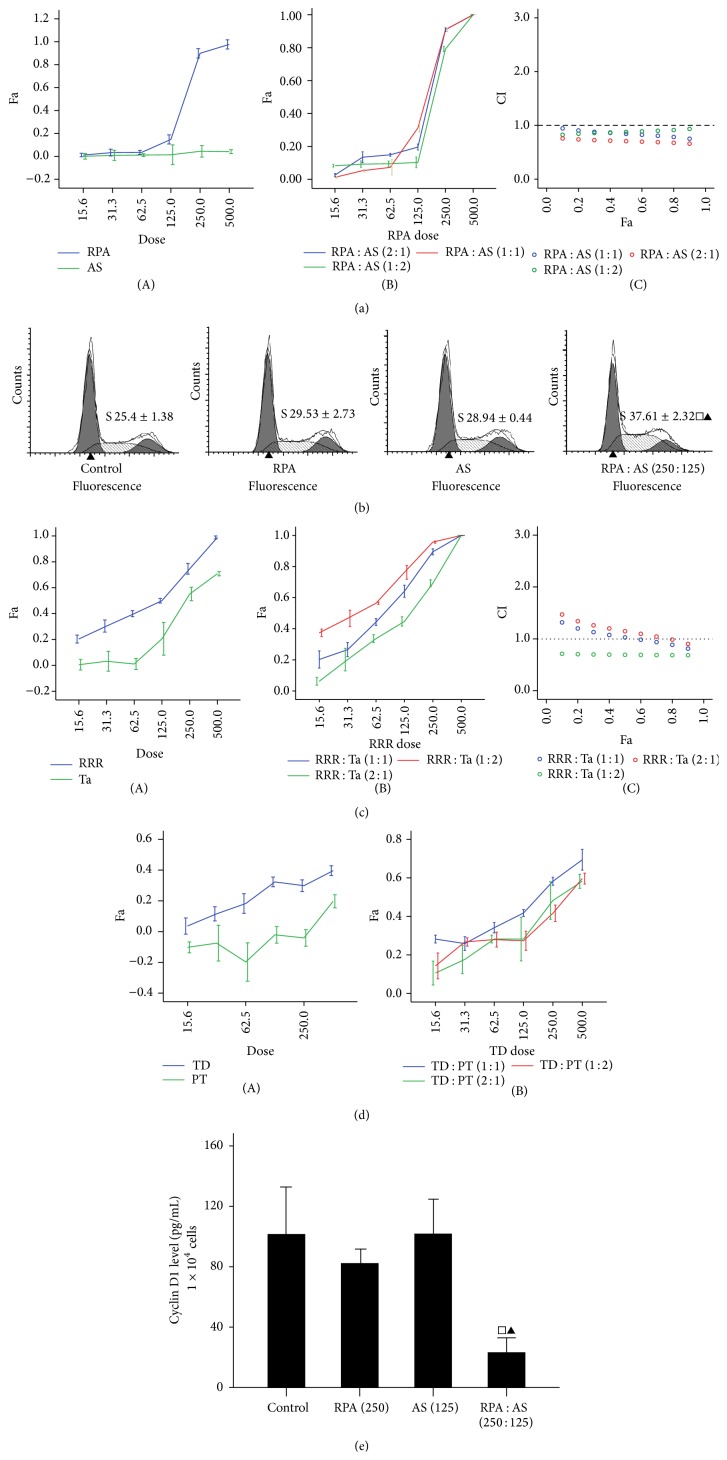
Median effect principle used to study the effect of Si Wu Tang, Di Dang Tang, and Shi Xiao San on HeLa cell. (a) Effect of Radix Paeoniae Alba (RPA) and* Angelica sinensis* (Oliv.) (AS) on HeLa cells. (A) Fa of cells treated with RAP or AS alone; (B) Fa of cells treated with a combination of RAP and AS; (C) CIs plotted for Fa. (b) Cell cycle distribution of HeLa cells treated with RAB and/or AS. Arabic numbers represent the percentage of cells distributed in the S phase. (c) Effect of* Radix* Rhizoma Rhei (RRR) and* Tabanus* (Ta) on HeLa cells. (A) Fa of cells treated with RRR or Ta alone; (B) Fa of cells treated with a combination of RRR and Ta; (C) CIs for Fa. (d) Effect of* Radix* et Trogopterus Dung (TD) and Pollen Typhae (PT) on HeLa cells. (A) Fa of cells treated with TD or PT alone; (B) Fa of cells treated with a combination of TD and PT. (e) Cyclin D1 level. □ Compared with control groups, *P* < 0.01. ▲ Versus RPA group, *P* < 0.05. Drug dose unit: *μ*g/mL.

The dose-effect curves of single or combined drug treatments were analyzed to test the effects of every combination of two drugs in Si Wu Tang (AS, RPA, LCH, and RRP) on cell proliferation by the median effect method. Our data showed that RPA effectively inhibited the proliferation of HeLa cells, whereas AS, LCH, and RRP had no significant influence. The CIs of the combined RPA and AS treatments at 1 : 1, 2 : 1, and 1 : 2 ratios were less than 1, showing that RPA and AS exert synergic effects. Moreover, the synergic effect was more pronounced when HeLa cells were treated with RPA combined with AS in a 2 : 1 ratio (250 *μ*g/mL : 125 *μ*g/mL) (Figure 3(a)).

RPA and AS did not impact the cell cycle distribution of HeLa cells. Interestingly, the percentage of S phase cells in the combined RPA and AS treatment group (250 : 125 *μ*g/mL) was much higher than those in the RPA or AS alone groups (Figure 3(b)). Moreover, RPA or AS alone did not influence the intracellular cyclin D1 content in HeLa cells. Dramatically, treatment with RPA and AS (250 : 125 *μ*g/mL) for 96 hours resulted in a significant decrease of intracellular cyclin D1 in HeLa cells, whereas RPA and AS alone did not influence the intracellular cyclin D1 content of HeLa cells (Figure 3(e)). AS did not induce apoptosis of HeLa cells, whereas 250 *μ*g/mL RPA and the concomitant treatment group did induce apoptosis. The apoptosis rates were 15.77 ± 1.36 (250 *μ*g/mL RPA group) and 10.17 ± 1.04 (250 *μ*g/mL RPA + 125 *μ*g/mL AS groups), with no significant difference (data not shown). Therefore, RPA and AS did not induce cellular apoptosis in a synergistic manner.

We used the same method to study the effect of Di Dang Tang and Shi Xiao San on the proliferation of HeLa. Our data showed that, in Di Dang Tang (RRR, Ta, SP, and Le), RRR effectively inhibited the proliferation of HeLa cells, whereas Ta (<250 *μ*g/mL), SP, and Le had no significant influence. The CIs of the combined RRR and Ta treatments at 1 : 2 were less than 1, showing that RRR and Ta exert synergic effects (Figure 3(c)).

Shi Xiao San is composed of the TD and PT. TD (>125 *μ*g/mL) alone inhibited the proliferation of HeLa cells. PT did not inhibit the proliferation of HeLa cells, but a low dose of PT (≤250 *μ*g/mL) promoted the proliferation of HeLa cells. Interestingly, when treated with TD and PT at 1 : 1, 2 : 1, and 1 : 2 ratios for 96 hours, the proliferations of HeLa cells were effectively inhibited. This inhibitory effect was most prominent when HeLa cells were treated with a combination of TD and PT at a 1 : 1 ratio (62.5~500 *μ*g/mL). The highest inhibition rate was 69.41% (Figure 3(d)).

### 3.3. Orthogonal Experimental Design Utilized to Diminish the Formulae

An L18 (5^3^) orthogonal array was utilized to determine the contribution of five positive herbs at three dose levels to determine the optimal combination. Data from the analysis of variance showed that, compared with the control group, the maximum inhibition rate was 99.5% in all combinations and that the minimum was 26%. There was a significant difference in inhibition of HeLa cells between the high and low dose groups for ER and CS (*P* < 0.05). This difference in the FAA group was not significant (*P* = 0.062), and, in the LJH and Tu groups, there was no difference (Table 2).

### 3.4. Comparison of Antitumor Activity In Vitro

**Figure 4:**
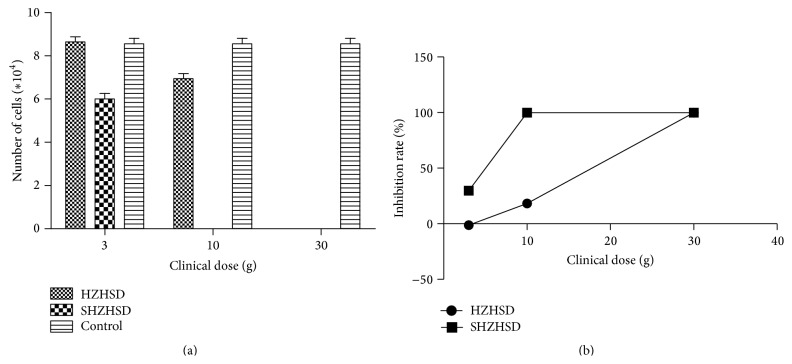
Effects of HZHSD and SHZHSD on the proliferation of HeLa cells. (a) Effects of HZHSD and SHZHSD on cell number. (b) Inhibition rates of HZHSD and SHZWSD on the proliferation of HeLa cells.

Through the experiments above, we identified the diminished smaller formula (SHZHSD) consisting of RPA, AS, RRR, Ta, TD, PT, ER, CS, and FAA from the original formula (HZHSD). The clinical dose of every herb was 6 g except that for RRR, which was 3 g. The results of the trypan blue exclusion assay showed that a clinical dose of only 30 g of HZHSD could significantly inhibit the proliferation of HeLa cells. However, clinical doses of 3 g, 10 g, and 30 g of SHZHSD effectively inhibited the proliferation of HeLa cells (Figure 4).

**Figure 5:**
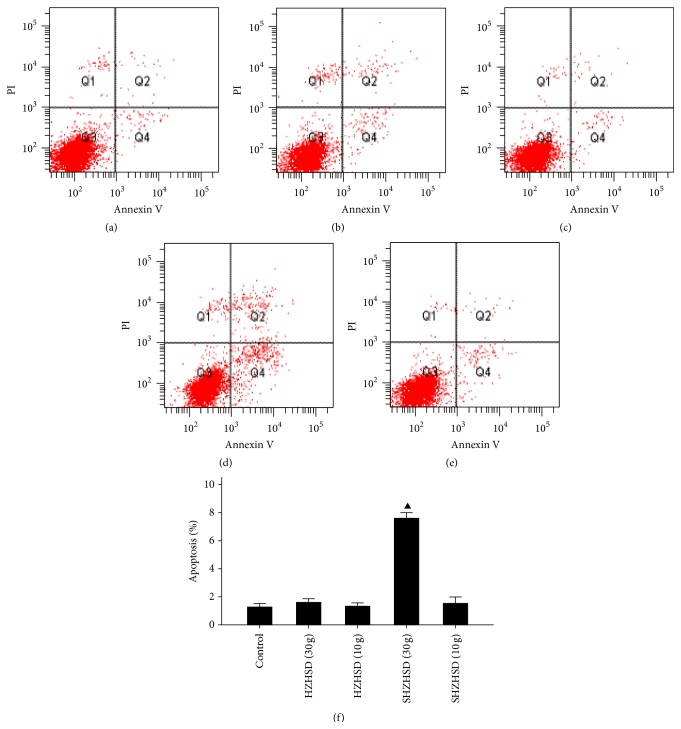
Effects of HZHSD and SHZHSD on cell apoptosis. (a) Control group. (b) HZHSD 30 g. (c) HZHSD 10 g. (d) SHZHSD 30 g. (e) SHZHSD 10 g. ▲ Versus control group, *P* < 0.01. HZHSD: Hua-Zheng-Hui-Sheng-Dan original formula. SHZHSD: diminished small formula consisting of 9 herbs from the original formula.

We carried out flow cytometry analysis on HeLa cells treated with HZHSD and SHZHSD. Our data showed that a clinical dose of only 30 g of SHZHSD induced apoptosis versus the control group. The apoptotic rate was 7.60 ± 0.26%. There might not be a biological significance for these small increases (Figure 5), which did not influence the cell cycle distribution of HeLa cells.

### 3.5. Comparison of Antitumor Activity In Vivo

**Figure 6:**
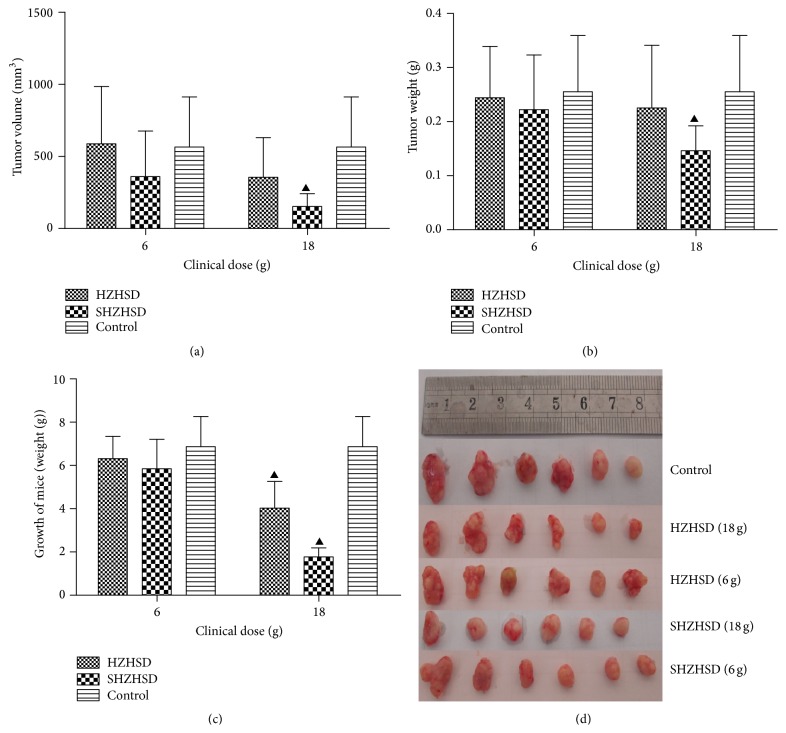
Effects of HZHSD and SHZHSD on the growth of mice (weight), tumor volume, and tumor weight. (a) Effects of HZHSD and SHZHSD on tumor volume. (b) Effects of HZHSD and SHZWSD on tumor weight. (c) Effects of HZHSD and SHZHSD on the growth of mice (weight). (d) Representative image of an excised tumor. ^▲^ *P* < 0.05 compared with control.

To determine the efficacy of HZHSD and SHZHSD in vivo, HeLa cells were implanted into the back of female nu/nu nude mice, and the growth of tumors was monitored at 3-day intervals. SHZHSD at a clinical dose of 18 g/d group was capable of significantly decreasing the tumor volume (Figure 6). The tumor weight in the groups treated with the clinical dose of 18 g/d SHZHSD was reduced by 42.75% (Figure 6). We measured the growth of mice (weight) and found that, compared with the control group, the administration of HZHSD and SHZHSD at 18 g/d reduced the weight of the mice. We consider that RRR caused diarrhea in these mice, which was the primary reason for the weight reduction.

## 4. Discussion

Combination therapy of TCM plays a more and more important role in the clinical treatment of tumor. Many studies demonstrated that combined TCM therapy could achieve better efficacy and survival benefit and relieve the adverse chemotherapy drug reactions [5, 19, 20]. One classical model of TCM combination therapy was called formulae, which has been advocated more than 2,500 years ago [21, 22]. Guided by TCM theories, formulae are always designed to consist of different types of herbs or minerals to improve the therapeutic efficacy. This complex prescription form limited the clinical application of formulae to a certain extent. Moreover, plenty of formulae consisted of large numbers of herbs or minerals rather than small number of ingredients. For this reason it is believed that, in formulae, multiple components could act on multiple targets and show synergistic therapeutic efficacies [21, 22]. For example, the Realgar-Indigo naturalis formula has been proven to be very effective in treating human acute promyelocytic leukemia [3]. Another Chinese medical formula, WRCP, has exerted its direct anticancer effect on both hepatoma cells in vitro and breast cancer in vivo [23–25].

However, very little research has been reported on large formulae consisting of large numbers of herbs or minerals. For large formulae, the precise mechanisms of function are difficult to understand and must be clarified using a modern approach. Aiming to explore a possible research method of analyzing large formulae, we conducted an investigation that incorporated modern molecular biology approaches and statistical analyses to study the effects of HZHSD treatments on human cervical cancer HeLa cells.

For the initial screening of the herbs, the antiproliferation effect of each herb on HZHSD was investigated. Our data showed that RRR, CS, ER, FAA, LJH, Tu, RPA, and TD effectively inhibited the proliferation of HeLa cells and SKOV3 cells. Among the 35 herbs in HZHSD, only RRR had an effect on the HeLa and SKOV3 cells viability. However, this method only studied single herbs separately by neglecting drug interactions. Therefore, to avoid the omission of positive herbs, we tried to research the interactions of herbs in three small formulae of HZHSD (Si Wu Tang, and Di Dang Tang and Shi Xiao San).

The median effect principle is widely used in drug combination studies. According to this method, the combinations of RPA with AS, and RRR with Ta, had a significant synergistic inhibitory effect on the proliferation of HeLa cells. By calculating CIs, the synergic effect was more pronounced when HeLa cells were treated with RPA combined with AS at a 2 : 1 ratio and RRR combined with AS at a 1 : 2 ratio. Because a low dose of PT (≤250 *μ*g/mL) could promote the proliferation of HeLa cells, we could not calculate the CIs of TD combined with PT. Interestingly, using the trypan blue counting method, we found that the proliferation of HeLa cells was effectively inhibited when treated with TD combined with PT. Therefore, using the median effect principle, we added three herbs (AS, Ta, and PT) to the original eight positive herbs.

Furthermore, we investigated the effects of RPA and/or AS on cell cycle distribution and apoptosis of HeLa cells. The combination of RPA and AS had a synergistic effect on inducing the cell cycle S phase arrest of HeLa cells. Cyclins help activate cyclin-dependent kinases, which phosphorylate downstream proteins that allow cells to progress through these checkpoints. Cyclin D1 is activated from the G1 phase to the S phase and from the S phase to the G2 phase [26]. RPA or AS alone did not influence the intracellular cyclin D1 content of HeLa cells. HeLa cells treated with RPA and AS (250 : 125 *μ*g/mL) for 96 hours caused a significant decrease of intracellular cyclin D1. However, they did not induce HeLa cell apoptosis in a synergistic manner.

Orthogonal experimental design is used in decomposed recipes of formulae. Compared with control group, all combinations were able to inhibit the proliferation of HeLa cells significantly. The inhibition rates ranged from 26% to 99.5%. However, there was no difference in the inhibitory effect in HeLa cells among the clinical doses of 0 g, 1 g, and 3 g for LJH (*P* = 0.713) and Tu (*P* = 0.333). Thus, LJH and Tu were considered unnecessary herbs.

Finally, we identified the diminished small formula of HZHSD (SHZHSD), which consists of RPA, AS, RRR, Ta, TD, PT, ER, CS, and FAA. A comparison of the antitumor activity between SHZHSD and HZHSD in vitro and in vivo showed that 3 g, 10 g, and 30 g clinical doses of SHZHSD effectively inhibited the proliferation of HeLa cells. In contrast, only the 30 g clinical dose of HZHSD could significantly inhibit the proliferation of HeLa cells. Furthermore, the 30 g clinical dose of SHZHSD induced apoptosis of HeLa cells. To determine the efficacy of HZHSD in vivo, a HeLa xenograft mouse model was used. SHZHSD at a clinical dose of 18 g/d significantly suppressed both tumor volume and tumor weight, which was reduced by 42.75%. Both HZHSD and SHZHSD at a clinical dose of 18 g/d reduced the weight of mice. We believe that diarrhea of the mice caused by RRR was the primary reason for this loss in weight.

Our research yielded few positive results concerning the antitumor mechanism of any single herb. We reviewed the relevant research concerning each herb in SHZHSD. Ogasawara and Yang found that evodiamine extracted from* Evodia rutaecarpa* had anti-invasive, antimetastatic effects and could reduce the apoptosis of carcinoma cells [27, 28]. APS-1d, a novel polysaccharide isolated from* Angelica sinensis*, exerts cytotoxic activity in several cancer cell lines in vitro [29].* Radix Paeonia alba* extract (RPAE) could induce apoptosis in HL-60 leukemic cells [30]. Sappanchalcone, a flavonoid extracted from* Caesalpinia sappan*, suppresses oral cancer cell growth and induces apoptosis through the activation of p53-dependent mitochondrial, p38, ERK, JNK, and NF-*κ*B signaling. Thus, it has potential as a chemotherapeutic agent for oral cancer [31]. The studies of Lin and Yaoxian et al. investigated that emodin, a natural anthraquinone derivative isolated from* Radix* et Rhizoma Rhei, had a potential antitumor effect on both pancreatic cancer cells and HeLa cells by its dual role in the promotion of apoptosis [32, 33]. It is demonstrated that the jaceosidin, isolated from* Artemisia argyi*, might be a potential drug for treating cervical cancers since this drug could inhibit the functions of E6 and E7 oncoproteins of the human papillomavirus [34]. However, we did not find reports concerning the antitumor effect of Ta, TD, or PT. Thus, the antitumor mechanism and targets of SHZHSD and drug interactions require further study.

In conclusion, this study demonstrates for the first time a possible research method of large formulae in TCM. We used human cervical cancer HeLa cells treated with HZHSD as a working model. Through molecular biology approaches in vitro and in vivo and by using the median effect principle and an orthogonal experimental design, we optimized and reduced the large formula. The small formula (SHZHSD) diminished from original formula (HZHSD) exhibited better antitumor activity than did the original formula in vitro and in vivo. SHZHSD exhibits this anticancer effect by inhibiting the proliferation and induction of apoptosis of HeLa cells.

## Figures and Tables

**Table 1:** 35 herbs in the large formula HZHSD.

Components of HZHSD	Dose (g)
*ginseng* (Ri)	180
Cortex Cinnamomi Cassiae (CC)	60
Radde Anemone Rhizome (RA)	60
*Moschus* (Mo)	60
Tumeric (Tu)	60
Flos Syzygii Aromatici (FSA)	90
Zanthoxylum Bungeanum Maxim (ZBM)	60
*Tabanus* (Ta)	60
Rhizoma Sparganii (RS)	60
Flos Carthami (FC)	60
*Caesalpinia sappan* (CS)	90
Semen Persicae (SP)	90
Fructus Perillae (FP)	60
Pollen Typhae (PT)	30
Trogopterus Dung (TD)	60
Lignum Dalbergiae Odoriferae (LDO)	60
Resina Toxicodendri (RT)	60
Radix et Rhizoma Rhei (RRR)	240
*Angelicasinensis* (Oliv.) (AS)	120
Myrrh (My)	60
Radix Paeoniae Alba (RPA)	120
Semen Armeniacae Amarum (SAA)	90
Nutgrass Galingale Rhizome (NGR)	60
*Evodiarutaecarpa* (ER)	60
Rhizoma Corydalis (RC)	60
Leeches (Le)	60
Asafoetida (As)	60
Fructus Foeniculi (FF)	90
*Ligusticum chuanxiong* Hort (LCH)	60
Frankincense (Fr)	60
*Alpihia officinarum* Hance (AOH)	60
Folium Artemisia Argyi (FAA)	60
*Leonurusjaponicus* Houtt (LJH)	240
Radix Rehmanniae Preparata (RRP)	120
Carapax Trionycis (CT)	500

**Table 2:** Stability studies of L18 (5^3^) orthogonal experimental design.

	Tu	ER	LJH	CS	FAA	Inhibition rate
1	0 g	0 g	0 g	0 g	0 g	0.0%
2	0 g	1 g	1 g	1 g	1 g	36.0%
3	0 g	3 g	3 g	3 g	3 g	99.5%
4	1 g	0 g	0 g	1 g	1 g	45.0%
5	1 g	1 g	1 g	3 g	3 g	87.0%
6	1 g	3 g	3 g	0 g	0 g	44.0%
7	3 g	0 g	1 g	0 g	3 g	26.0%
8	3 g	1 g	3 g	1 g	0 g	32.0%
9	3 g	3 g	0 g	3 g	1 g	95.9%
10	0 g	0 g	3 g	3 g	1 g	84.6%
11	0 g	1 g	0 g	0 g	3 g	43.4%
12	0 g	3 g	1 g	1 g	0 g	55.0%
13	1 g	0 g	1 g	3 g	0 g	80.1%
14	1 g	1 g	3 g	0 g	1 g	37.0%
15	1 g	3 g	0 g	1 g	3 g	85.0%
16	3 g	0 g	3 g	1 g	3 g	61.7%
17	3 g	1 g	0 g	3 g	0 g	83.1%
18	3 g	3 g	1 g	0 g	1 g	44.9%
*P*	0.333	0.024	0.713	<0.001	0.062	

*P*: significance of the difference between the three levels (low dose, high dose, and control group) of each factor.

Tu: Tumeric; ER: *Evodiarutaecarpa*; LJH: *Leonurusjaponicus* Houtt; CS: *Caesalpinia sappan*; FAA: Folium Artemisia Argyi.
